# Styptic Effect of Kreasote

**Published:** 1842-11

**Authors:** 


*Styptic Effect of Kreasote.*—A robust countryman divided the ulnar artery with a sharp knife, the consequence of which was repeated bleedings, which, however, were staunched by surgical aid. Three weeks afterwards, hemorrhage returned, and Dr. Burdach of Luckan was sent for. He found the wound, which at first was a simple puncture, livid at the edges, and expanded to the size of the palm of the hand, by a spongy growth from the bottom. This spongy mass was in a gangrenous condition, and prevented the examination of the wound; the arm was swollen from the shoulder to the finger points; it could not be moved, and was excessively painful. Dr. Burdach had only the choice between actual cautery and kreasote left, for in such a state of the arm, tying the artery was out of the question. He poured ℥ss of kreasote (*freed from eupion*) into the wound. This gave the patient no pain; nay, after it he enjoyed refreshing sleep for the first time after the accident. There was no more hemorrhage; the pouring in of the kreasote was repeated morning and evening, and the spongy mass gradually diminished, and three days afterwards, under the co-operating influence of bandages moistened with kreasote, ol tereb., and balsam. indic., loosened itself from the bottom of the wound. The divided artery was no more visible, the swelling of the arm decreased, and the complete cure shortly followed without any relapse.—*Lond. and Edin. Monthly Jour. Med. Sci.*, May 1842, from *Medicinische Zeitung*, Jahrg. 1840. No. 31.

